# The prognostic outcome of ‘type 2 diabetes mellitus and breast cancer’ association pivots on hypoxia-hyperglycemia axis

**DOI:** 10.1186/s12935-021-02040-5

**Published:** 2021-07-05

**Authors:** Ilhaam Ayaz Durrani, Attya Bhatti, Peter John

**Affiliations:** Atta-ur-Rehman School of Applied Biosciences (ASAB), National University of Sciences and Technology (NUST), H-12, Islamabad, Pakistan

**Keywords:** Breast cancer, Type 2 diabetes mellitus, T2DM, Hypoxia inducible factor 1, HIF1, Hypoxia, Hyperglycemia

## Abstract

Type 2 diabetes mellitus and breast cancer are complex, chronic, heterogeneous, and multi-factorial diseases; with common risk factors including but not limited to diet, obesity, and age. They also share mutually inclusive phenotypic features such as the metabolic deregulations resulting from hyperglycemia, hypoxic conditions and hormonal imbalances. Although, the association between diabetes and cancer has long been speculated; however, the exact molecular nature of this link remains to be fully elucidated. Both the diseases are leading causes of death worldwide and a causal relationship between the two if not addressed, may translate into a major global health concern. Previous studies have hypothesized hyperglycemia, hyperinsulinemia, hormonal imbalances and chronic inflammation, as some of the possible grounds for explaining how diabetes may lead to cancer initiation, yet further research still needs to be done to validate these proposed mechanisms. At the crux of this dilemma, hyperglycemia and hypoxia are two intimately related states involving an intricate level of crosstalk and hypoxia inducible factor 1, at the center of this, plays a key role in mediating an aggressive disease state, particularly in solid tumors such as breast cancer. Subsequently, elucidating the role of HIF1 in establishing the diabetes-breast cancer link on hypoxia-hyperglycemia axis may not only provide an insight into the molecular mechanisms underlying the association but also, illuminate on the prognostic outcome of the therapeutic targeting of HIF1 signaling in diabetic patients with breast cancer or vice versa. Hence, this review highlights the critical role of HIF1 signaling in patients with both T2DM and breast cancer, potentiates its significance as a prognostic marker in comorbid patients, and further discusses the potential prognostic outcome of targeting HIF1, subsequently establishing the pressing need for HIF1 molecular profiling-based patient selection leading to more effective therapeutic strategies emerging from personalized medicine.

## Introduction

Diabetes and cancer are two complex yet increasingly prevalent, chronic, metabolic morbidities [1], emerging globally as major public health concerns [2]. Diabetes with a global occurrence of 422 million in 2014 [3], is the seventh leading cause of death worldwide [4], while cancer is undisputedly the second most leading cause of death with 17 million cases in 2018 alone and an expected 27.5 million new cases by 2040 [5], its prevalence is set on ever increasing.

Diabetes itself is a group of various metabolic disorders, characterized by hyperglycemia resulting from insulin deficiency, its inaction or both, with a spectrum of other complications affecting major body organs associated with it. It can be classified into type 1 and type 2 diabetes mellitus and gestational diabetes [6]. Type 1 diabetes mellitus (T1DM) is insulin dependent and caused by the inability of the pancreatic beta cells to synthesize insulin, accounted for by the autoimmune destruction of beta cells [7]. Type 2 diabetes mellitus (T2DM), on the other hand is not insulin dependent; rather it is characterized by insulin resistance due to the inability of cells to respond to insulin followed by compensatory hyperinsulinemia [8]. Cancer too is a heterogeneous disease and can be classified into many distinct types based on its site of origin and other clinic-pathological features and molecular signatures [9, 10].

There have been many speculations since the twentieth century, linking diabetes to cancer and increasing evidence has associated diabetes, particularly type 2 diabetes mellitus (T2DM) with cancer risk, prognosis and treatment. These are supported by numerous epidemiological studies that revealed a positive correlation of T2DM with many types of cancer, such as pancreas [11], liver [12], endometrium [13], colon-rectum [14], bladder [15], and breast cancer [16].

However, it is noteworthy that not all cancers associate positively with diabetes. Prostate, kidney and ovarian cancers are inversely associated with diabetes i.e. diabetic patients are at a decreased risk of developing these cancers [17], and lung cancer has reportedly shown no association with either type of diabetes.

Cancers of liver, endometrium and pancreas have shown doubled risk as compared to breast, colon, rectal and bladder cancers in T2DM patients. Furthermore, cancers that associate with one type of diabetes may not necessarily also associate with the other type, such as breast cancer which only associates with T2D and not with T1D [18]. Insulin and other drugs have also been associated with the diabetes-cancer risk; however, the debate is still ongoing [19–24]. Hence, with review emerges complexities in the link between diabetes and cancer and the multi-faceted relationship these two diseases hold. The increase in cancer risk in diabetic patients may be slight to moderate, yet given the status of diabetes as a global epidemic, the socioeconomic impact of this positive association may be significantly burdening [25].

Following the discovery of the hallmarks of cancer [26, 27], the metabolic nature of some of the deregulations involved in cancer has become a focus of study worldwide and these may be in line with the metabolic abnormalities’ characteristic of diabetes. Yet, the root cause of this diabetes- cancer association is still under explored and hence not fully clear. There have been multiple possible molecular mechanisms proposed to explain the causal relationship [28]. The more extensively researched and hence the better understood hypotheses include hyperinsulinemia and hyperglycemia [29]. These along with inflammation and oxidative stress related conditions may be of prime importance in explaining the link and hence these interconnected states would be reviewed further in this article.

The etiology underlying these two diseases and the interplay of common pathways may help to explain the molecular basis of the positive correlation. Diabetes involves metabolic alterations, hormonal imbalances particularly of insulin/insulin growth factor-1 and adiponectin/ leptin, and immune response such as elevated levels of pro-inflammatory cytokines like tumor necrosis factor-a (TNF-α), and all these features it shares with cancer. These commonalities may give insight into the cross talk that initiates carcinogenesis in diabetic patients and may reflect on a cumulative effect of the converging and diverging pathways in combination with the differential pathways modulating the two-way relationship between diabetes mellitus and cancer; of diabetes as a cause and as an aftermath of cancer. Additionally, both these diseases share certain risk factors including diet, exercise, ageing and obesity [30], hence these and the reported crosstalk within the insulin and cancer signaling pathways not only spark an interest in elucidating the correlation between diabetes and cancer but it may also be employed to understand the connection.

Hyperinsulinemia may be a direct causal factor for carcinogenesis. Findings from numerous studies have consistently linked hyperinsulinemia, which is characteristic of type 2 diabetes mellitus with increased incidence of cancer [31, 32]. Insulin has mitogenic properties and may lead to cancer initiation itself and via increasing Insulin-like growth factor (IGF-1), which has both mitogenic and anti-apoptotic properties.

 Furthermore, hyperglycemia is a hallmark of diabetes [33], and is also a risk factor for cancer progression [34]. It has shown to trigger the HIF1 pathway via up-regulation of HIF1-α gene expression, eventually leading to an anti-apoptotic cell response and activation of oncolytic pathways in specific cell types [35–37]. A study conducted on rat pancreatic beta cells reported high glucose induced increased oxygen consumption, leading to hypoxia and subsequent HIF1 activation, and associated it with a slower decrease in beta cell function [38]. In another instance, hyperglycemia mediated HIF1 activation was also correlated with glucose intolerance [39]. However, there are studies showing that hyperglycemia in diabetic patients impair the HIF pathway [35], differing with studies which show an increased expression of HIF1-α under hyperglycemia [40]. Hence, the critical role of HIF1 in establishing the diabetes-cancer link needs to be fully elucidated by probing into the intricate crosstalk between HIF1 and insulin signaling.

Other proposed potential biological mechanisms include hormonal imbalances disrupting the estrogen-progesterone and adiponectin/lectin axes and also obesity as an independent risk factor [41–44]. Still, the current knowledge pertaining to the exact nature of the molecular mechanisms linking diabetes with cancer and what is currently known only gives a very complex picture of status quo. Latent cancer may also contribute to the development of diabetes and hence lead to reverse causality [45]. Either way, co-occurrence of diabetes and cancer seems to worsen the prognosis and increase mortality [46, 47].

This review however focuses on T2DM- breast cancer association by discussing disease epidemiology, pathogenesis and underlying potential molecular mechanisms establishing a crosstalk between diabetes and breast cancer signaling pathways and highlights on the relevance of hypoxia-hyperglycemia axis established, in explaining this molecular association. It presents an overview of series of events that may initiate carcinogenesis in mammary tissue and contribute to its progression in diabetic patients, outlining the plausible molecular routes from diabetic state to breast carcinogenesis, with a particular emphasis on the role of HIF1 in mediating this transition in diseased state. It further discusses the outcome of this association on breast cancer subtype specificity, and relates this dynamic molecular crosstalk to explain the development of hallmarks of cancer, Additionally, this review establishes on the multi-faceted, bidirectional and dual relationship between diabetes, its treatment and breast cancer subtype specific incidence, prognosis and therapy.

Breast cancer was specifically selected as an area of focus, as it shares several characteristics with T2DM, including its characterization by regions of hypoxia, its composition of adipose tissue, a major site affected by T2DM, and the high incidence of breast cancer, in Pakistan, and worldwide.

Understanding the diabetes- breast cancer link on hyperglycemia-hypoxia axis may give way to understanding the role of HIF1 in developing this link, which could then be employed to determine the prognostic outcome of targeting HIF1 in comorbid patients. This review may also inspire further research and identification of potential biomarkers for early detection of breast cancer in diabetic patients and prognosis prediction in comorbid patients.

## Literature review article selection

**Fig. 1:**
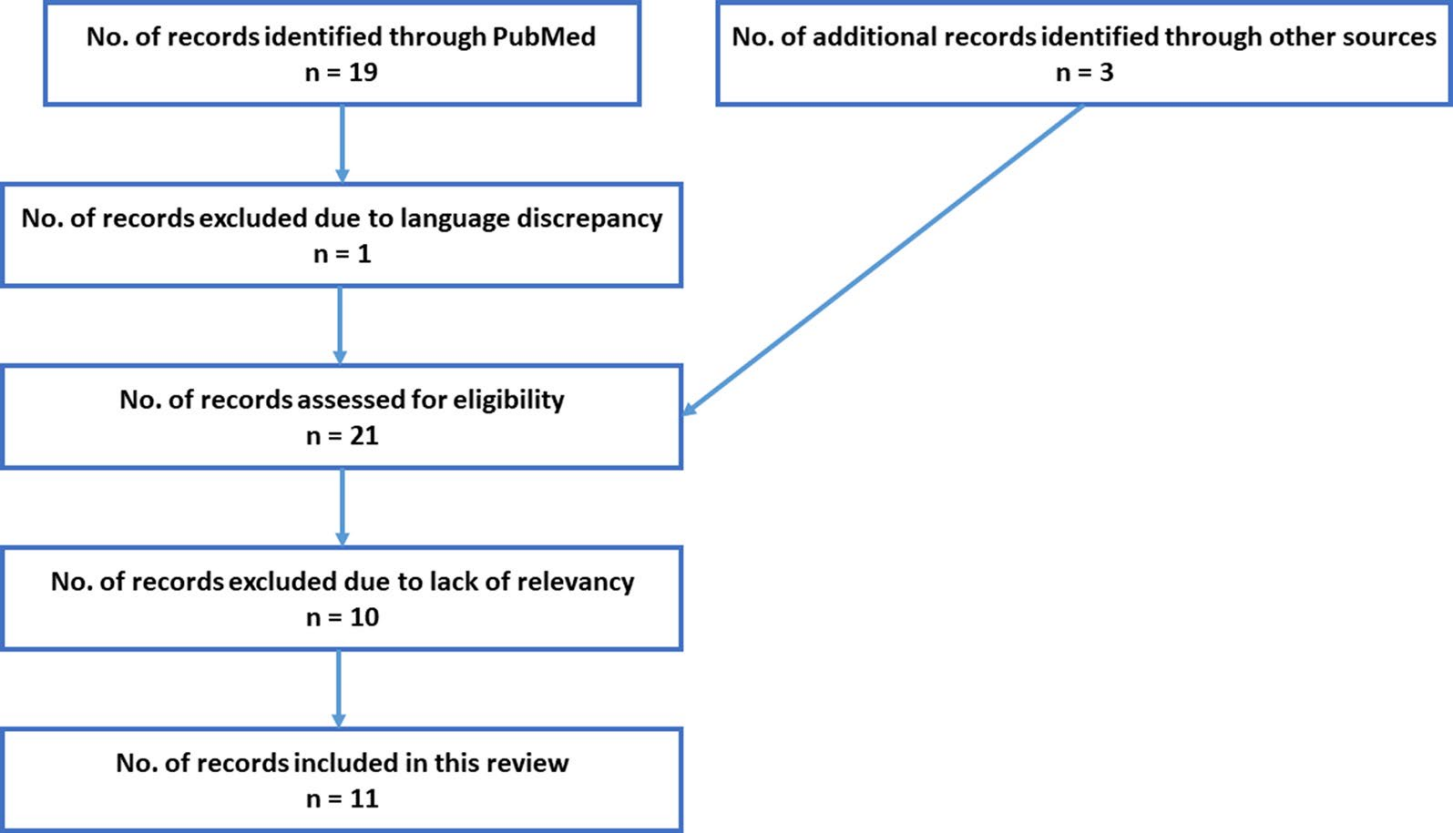
Article selection process outline. Out of 21 articles matching the keywords search results, only 11 matched the inclusion criteria and hence were included in this review

For this literature review, the online database PubMed was searched through using the keywords “diabetes” (OR “T2DM” OR type 2 diabetes mellitus) AND “breast cancer” (OR “breast carcinoma”) AND “hypoxia inducible factor 1” (OR “HIF1”) in all possible combinations. A total of 19 articles were extracted for the period 2008–2021, of which only 9 matched the relevancy of all three keyword categories inclusion criteria. Of these 1 article was in Chinese, hence could not be fully reviewed here. Out of the remaining, 6 discuss the role of potential therapeutics targeting diabetes and breast cancer, while also implicating HIF1-α. Additionally, 3 articles were extracted from other searches such as studying review articles to extract references for original articles relevant to the research topic as outlined in Fig. 1 enlisting the article selection process. For this review, 11 articles were eligible for inclusion and are discussed henceforth.

## Epidemiological links of diabetes with breast cancer

Breast cancer is the fifth most common cause of death [48], and the most commonly diagnosed cancer in women [49]. There were over 2 million cases reported in 2018 alone. Pakistan has the highest breast cancer incidence rate in Asia, with approximately 90,000 cases reported every year and 40,000 deaths. It is reported that one in every nine Pakistani women is likely to develop breast cancer at some stage in her life [50].

Numerous studies have linked diabetes with a small yet increased breast cancer risk and even worse prognosis as summarized in Table 1. So far, this has been attributed to insulin resistance and associated corresponding hyperinsulinemia. For instance, studies tracking death in breast cancer patients reported an increasing trend for women with diabetes, and also supported a stronger independent link with prognosis as compared to breast cancer risk [51], which can be explained by higher insulin levels, late diagnosis, less aggressive treatment plan or diabetes associated complications or co-morbidity [52, 53].

**Table 1:** Epidemiological data linking diabetes with breast cancer

Sr. No.	Study name	Country	Study type	Population/Sample size	Study period	Age group	Characteristic findings	Refs
1	Nurse Health Study	USA	Follow up	116,488 Nurses	1976–1988	30–55	Women with T2DM had a modestly elevated breast cancer incidence	[54]
2	Long Island Breast Cancer Study Project	USA	Population based study using data from case–control & Follow up studies	1508	1996–1997	30 +	Diabetes associated increased breast cancer incidence in older and non-white women due to all reasons	[55]
3	SEER-Medicare based study		Observational Cohort	2418	2001–2007	> 80 Mean age: 77.8	Diabetes associated with advanced cancer stage and increased mortality	[56]
4	Meta-analysis of diabetes mellitus and risk of breast cancer	Various (From North America, Europe & Asia)	Meta-analysis of case–control &cohort studies	20 Studies (30,568 cases)	1966–2007	20–95	20% increased breast cancer risk in women with diabetes	[17]
5	Retrospective cohort study in China	China	Retrospective Cohort	36 cases	2002–2008	-	Increased risk of developing breast cancer in T2DM patients	[57]
6	Diabetes increases risk of breast cancer	Various	Meta-analysis of case–control & cohort studies	43 studies (422,631 cases)	Oldest Study from 1990 Latest from 2012	Varied	T2DM increases the risk of breast cancer in women	[58]
7	Random effects model based meta-analysis	Various (From North America, Europe & Asia)	Meta-analysis	39 independent observational studies (58,690 cases)	Oldest Study from 1993 Latest from 2011	All ages	27% Increased risk for breast cancer in women with T2DM (reduced to 16% after adjustment for BMI)	[18]
8	T2DM as a risk factor for female breast cancer	Pakistan	Case–control study	400 patients	2014–15		17.69% breast cancer patients reported diabetes	[59]

Data represented here has been retrieved from 7 published works including 3 meta-analyses and other population based studies

Another follow up study of 116,488 female nurses revealed 6220 women with T2DM, and 5189 incident cases of invasive mammary carcinoma in the course of time. A modestly elevated risk for breast cancer incidence in T2DM patients was reported, independent of the influence of other risk factors including age, obesity and family history, physical activity and reproductive factors [54].

T2DM as a prognostic factor in breast cancer patients has also been associated with decreased overall survival. A cohort study of one million U.S adults revealed a 16% increase in breast cancer mortality in patients with pre-existing diabetes [60]. This was consistent with multiple other studies reporting a higher all-cause mortality in diabetic women [61]. Hence co-occurrence of diabetes with breast cancer may worsen the prognosis and overall survival by increasing the risk of wound infection and hence subjection to a delay in administration of adjuvant chemotherapy. This may contribute to worsening of the patient’s state [62]. Diabetic treatment may also interfere with disease outcome and patient survival. Studies have linked it to distant metastases and increased recurrence rates in breast cancer [63].

Hence, another layer of complexity arises as anti-diabetic treatments have also shown to impact breast cancer risk and prognosis. Insulin sensitizers such as metformin decreases insulin resistance by altering the PI3K/AKT/mTOR pathway [64]. This may potentially contribute to the anti-cancer effects of metformin such as a decrease in cell proliferation and induction of apoptosis when tested on cancer cell lines [65]. A recent population based case-case study in 2019, evaluated the association between T2DM, its medications and breast cancer risk, particularly in different molecular subtypes [66]. The study involved 4557 breast cancer patients, and showed that women with T2DM had a 38% increased risk of triple negative breast cancer, as compared to women lacking T2DM history, and particularly for those prescribed with metformin for long-term usage. On the other hand, in the Women’s Health Initiative study, metformin use was found to correspond with a lower risk of hormone receptor positive and HER2 negative breast cancer [67]. Contrary to this, epidemiological analysis based on the Black Women’s Health Study cohort, reported no significant differences for breast cancer risk based on ER status, diabetic condition and diabetic treatment [68]. The inconsistency in these findings may be explained by a potential misclassification bias originating from self-reported data on metformin use, and even by the overall prevalence of diabetic medication amongst the study population. Moreover, metformin’s role as an adjuvant in chemotherapy for breast cancer has also been reviewed [69].

Other diabetic treatments include externally administrated insulin, which is known for its mitogenic effects [70], and has been associated with a non-significant increased risk of breast cancer [71]. While, insulin glargine is associated with 30% increased risk [72], and meta-analyses investigating breast cancer risk associated with sulfonylurea use have also yielded mixed results, lacking consistency [73–75], and hence further research is needed to substantiate the association between diabetic treatments and breast cancer risk.

Cancer treatment may also contribute to the development of diabetes, however breast cancer in patients with pre-existing diabetes has shown increased disease severity [76]. The type of breast cancer treatment is also known to influence the incidence of T2DM in breast cancer patients [77]. In case of patients subjected to chemotherapy/surgery, 17.2% reported the development of T2DM [78]. Patients who underwent adjuvant chemotherapy, hormone therapy, aromatase/PI3K inhibitors and morphine users showed an increased risk of developing T2DM [79–82]. Additionally, post-menopausal breast cancer is also shown to associate with higher T2DM incidence. Hence, the co-existence of diabetes with breast cancer may influence breast cancer prognosis and its treatment strategy as well.

Apart from insulin itself, several other nodes from insulin signaling have been implicated in breast cancer. (IGF) IGF binding proteins (-BP) regulate the bioavailability of IGF1 and IGF2, with IGFBP2 and IGFBP3 most abundantly circulating in blood [83]. Meta-analyses reveal that IGFBP2 and IGFBP3 both correlate positively with breast cancer and poor prognosis in patients [83, 84]. Hence, the multi-layered relationship between T2DM and breast cancer needs further probing into to fully comprehend the complexities of this bi-directional association.

## Prevalence, risk factors and mutual features of diabetes and breast cancer

### Etiology of diabetes

Diabetes is a complex metabolic syndrome that includes metabolic and hormonal disorders such as type 1 diabetes mellitus (T1DM), type 2 diabetes mellitus (T2DM) and gestational diabetes. A prolonged existence of hyperglycemic conditions in diabetic patients is associated with organ damage, failure and dysfunction, particularly of kidneys, heart, eyes, nerves and blood vessels [85].

The vast majority of diabetes cases fall into type 1 and 2 diabetes mellitus, where the latter being significantly more prevalent. T1DM is caused by an absolute lack of insulin secretion due to the autoimmune mediated destruction of beta cells in pancreas, which secrete insulin. This form of diabetes contributes to 5–10% of the total reported cases. T2DM, on the other hand is attributed to a combination of insulin resistance and a lack of / inadequacy of insulin secretory response. T2DM accounts for ~ 90–95% of the reported cases of diabetes [86]. The risk of developing T2DM is associated with factors such as age, obesity, and lack of physical activity. The genetics of this type of diabetes is complex and still not clear.

Type 2 diabetes originates in various sites including adipose tissue which becomes insulin resistant caused by alterations in the insulin signaling pathway [87], and so it is important to understand the pathway that directly impacts diabetes.

### Etiology of breast cancer

**Fig. 2:**
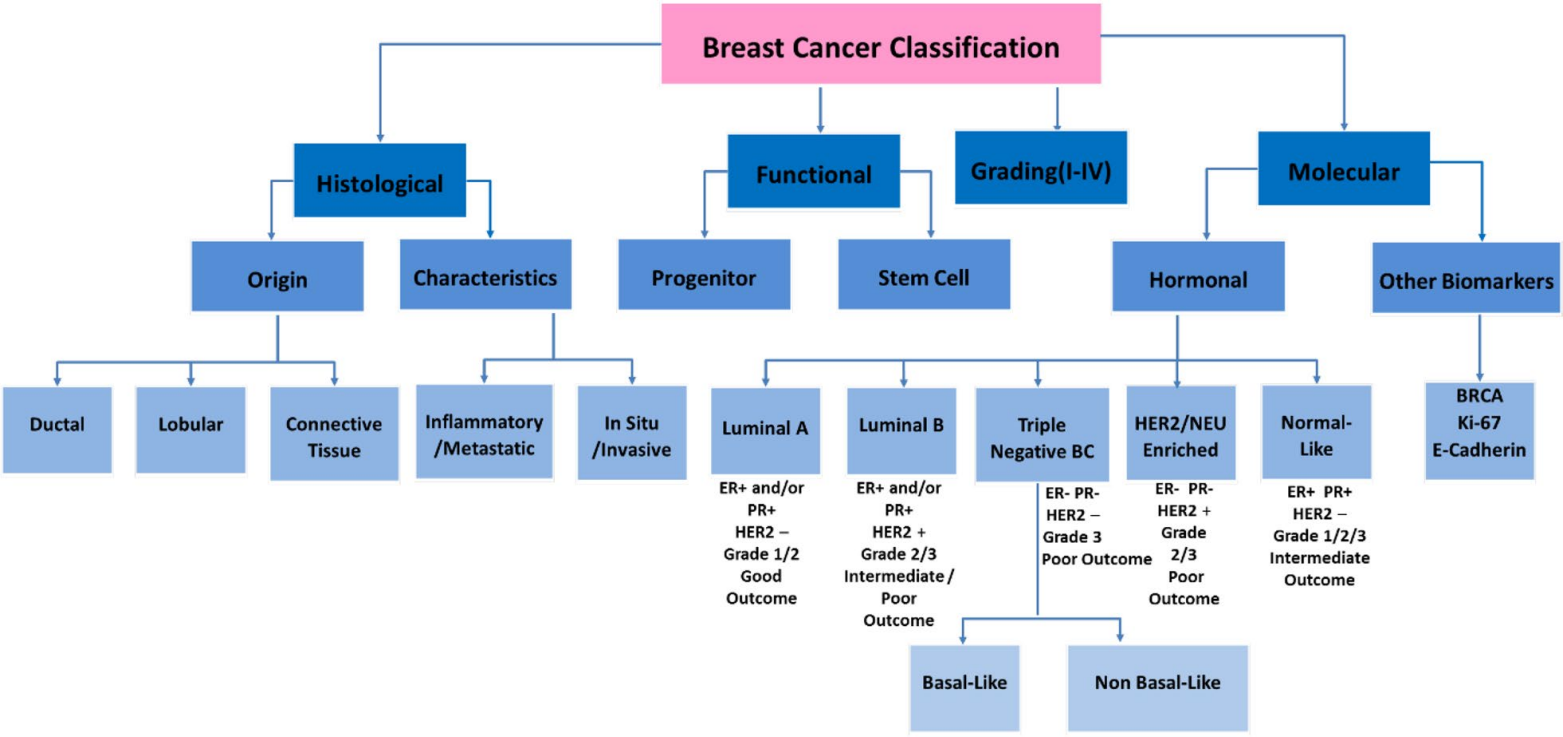
Breast cancer classification. The figure illustrates categories based on which breast cancer is sub-typed including histological, functional, grading and molecular biomarker expression [91–99]

Breast cancer originates in breast tissue which is composed mainly adipose and fibroglandular tissue, supporting lobules containing milk producing glands and ducts linking glands to the nipple [88, 89]. It is a complex, heterogeneous disease and presents a diverse risk profile. Even though all the causes of breast cancer are not fully understood, some of the risk factors include genetic mutations deregulating crucial signaling pathways and activation of oncogenes, increased body weight and obesity, prolonged exposure to carcinogens and alterations in the immune conditions which may promote cancer growth [90]. Breast cancer can be further classified into different groups based on their hormone receptor profiles and other categories as summarized in Fig. 2.

### Adipose tissue: common ground for T2DM and breast cancer

Adipose tissue is one the major sites affected by the development of T2DM. Deregulation in the insulin-AKT pathway leads to the development of the insulin resistance in adipocytes. Interestingly, it also forms a major and crucial component of breast tumor microenvironment, and is involved in major energy storage and glucose expenditure of the body [89, 100]. It also serves as an endocrine organ, secreting adipokines such as leptin and adiponectin, cytokines like interleukins and TNF-α, chemokine CXCL-8 amongst others, growth factors including but not limited to vascular endothelial growth factor (VEGF) and IGF, and other factors such as aromatase, all of which are associated with breast cancer [98–100].

### Metabolic reprogramming

Molecular alterations pertaining to diabetic phenotype such as defects in insulin signaling leading to lack of response to insulin and subsequent hyperinsulinemia, may ultimately promote tumorigenesis via HIF1 regulation, increased glucose uptake and its utilization, making energy more readily available to afford the uncontrolled growth of tumor [101]. Once HIF1 is activated, it increases the transcriptional expression of metabolic enzymes such as lactate dehydrogenase (LDH), and pyruvate dehydrogenase kinase (PDK1) which blocks the conversion of pyruvate to acetyl-coA, hence preventing pyruvate molecules from entering the TCA cycle.

## Signaling cascades and their crosstalk in diabetes and breast cancer

### Insulin signaling

Insulin is a peptide hormone released in response to high blood glucose level by pancreatic beta cells and it stimulates increased uptake of glucose by cell, glycogen synthesis in liver, gluconeogenesis, protein catabolism and fatty acid esterification on binding its receptor and initiating downstream signaling. It also inhibits lipolysis, autophagocytosis, proteosomal activity and apoptosis. Elevated insulin levels are associated with mitogenic effects in synergy with increased bioavailable IGF-1 levels.

Insulin/IGF1 signaling pathway controls metabolism and growth. Both insulin and IGF1, two key players required to trigger this pathway act on two closely related tyrosine kinases which when phosphorylated initiates a series of further phosphorylation events that regulate metabolic and cell growth pathways. Alterations in this network can lead to insulin resistance and diabetes [102].

Insulin and IGF-1 bind to and lead to a conformational change in insulin and IGF-1 receptors, respectively, following auto-phosphorylation. This leads to the recruitment and phosphorylation of receptor substrates such as insulin receptor substrate (IRS) and Shc proteins. IRS then recruits PI3K, and hence activates the AKT-PI3K pathway, which controls cell survival and growth. AKT also regulates insulin-mediated response including glucose transport, gluconeogenesis, glycogen synthesis and lipid synthesis. Shc activates the MAPK pathway, which then mediates cellular proliferation and transcription.

Hence, insulin and its signaling pathway play a crucial role in energy homeostasis by regulating glucose and lipid metabolism and by acting directly on liver, skeletal muscle and adipose tissue predominantly. Lack of insulin or its inadequacy in cases of diabetes leads to alterations in the pathway, which greatly influence the disease prognosis.

### HIF1 signaling

Tumor hypoxia is a driving force for metabolic alterations in cancer. Hypoxic conditions especially in solid tumors such as breast cancer trigger HIF1 signaling, which plays a central role in mediating cell's adaptive response to hypoxia.

HIF1 is a transcription factor belonging to the HIF family, regulated by both hypoxic and non-hypoxic conditions. It is a hetero-dimer with the two subunits constitutively produced [103]. However, under normoxic conditions, HIF1-α is proteosomally degraded after being hydroxylated by oxygen sensing PHD and FIH-1 enzymes. If the oxygen levels fall, the HIF1-α subunit is not hydroxylated, hence leading to its stabilization and binding to HIF1-β subunit to activate the downstream HIF1 signaling pathway [104].

Hypoxia independent activation of HIF1 pathway under normoxia has been shown to be triggered by signaling molecules such as nitric oxide, interleukin 1 (IL-1), tumor necrosis factor alpha (TNF-α), angiotensin II and growth factors such as epidermal growth factors, insulin and insulin like growth factor and also mediated by PI3K-AKT pathway [105, 106].

P53 regulates HIF1-α stability whereas the ERK/MAPK pathway regulates both HIF1- α stability and its activation; ERK is involved in the transcriptional activation and synthesis of HIF1-α [107]. PI3K-AKT pathway also regulates HIF1 protein translation via the action of mTOR on other genes controlling HIF1-α protein translation. Additionally, IL-1β also leads to HIF1 signaling in case of an inflammation. HIF1 in return, increases the entry of glucose into the cells, the glycolytic flux and the conversion of pyruvate into lactate [108].

**Fig. 3:**
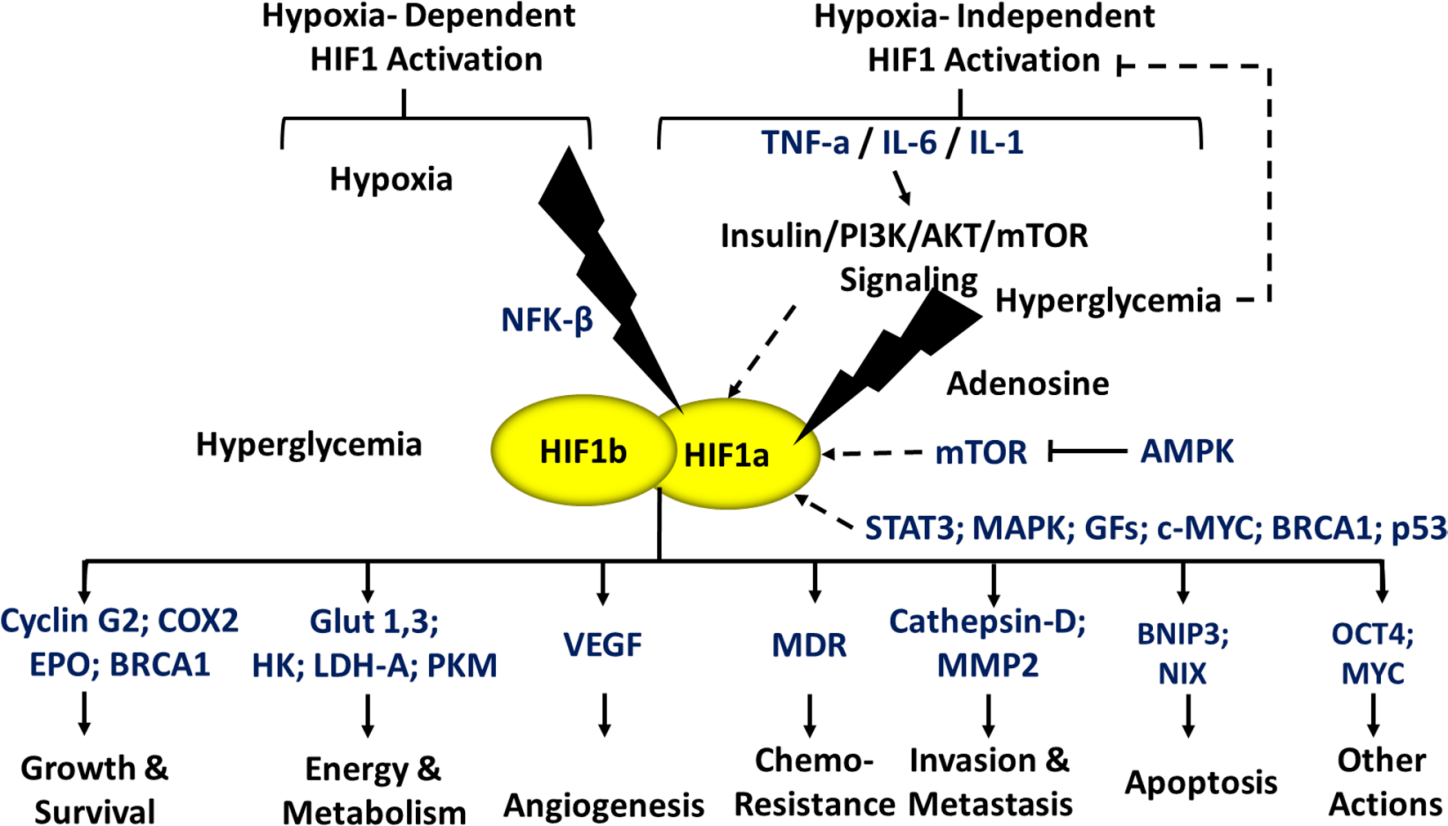
HIF1 signaling. HIF1 is a dimeric transcription factor composed of an oxygen concentration sensitive HIF1-α subunit and a constitutively present HIF1-β subunit. Under hypoxic conditions, HIF1-α stabilizes and forms a dimer with HIF1-β subunit to transcriptionally activate the expression of HIF1 target genes. HIF1 has also been shown to be activated under normoxic conditions by various factors as depicted [108–116]

Additionally, the hypoxia-independent regulation of HIF1 is mediated by signaling such as the insulin-PI3K-AKT, MAPK/ERK, IL-1 and NF-κB pathways. Once HIF1 α-β complex forms, it transcriptionally activates various target genes including glucose transporter GLUT1 and glycogen synthase kinase (GSK) which regulate metabolism, and e- cadherin and matrix metalloproteinase (MMP) proteins leading to epithelial to mesenchymal transition and metastasis. It also regulates autophagy and cell death via BCL2/adenovirus E1B 19 kDa protein-interacting protein (BNIP3) and p53, and angiogenesis by upregulating VEGF and MMPs as represented by Fig. 3.

### PI3K/AKT and ERK/MAPK pathways

AKT-PI3K pathway crucial for cell survival [117], and the ERK/MAPK pathway with its role in cell proliferation, converge at HIF1 signaling [118, 119], after diverging from insulin pathway [120, 121]. Both these pathways are central to cancer signaling and have been thoroughly reviewed elsewhere [122, 123], however it is important to relate to all these signaling cascades together to completely envision the complex picture within a developing cancerous cell.

### Pathways that crosstalk

Cancer being a complex phenomenon involves an intricate merger of various signaling cascades including but not limited to the pathways already discussed, yet this paper aims to elucidate on the central role of HIF1 signaling in mediating the crosstalk and disease state. Tracing its role in diabetes may give insight into the molecular basis for diabetes-associated breast carcinogenesis.

**Fig. 4:**
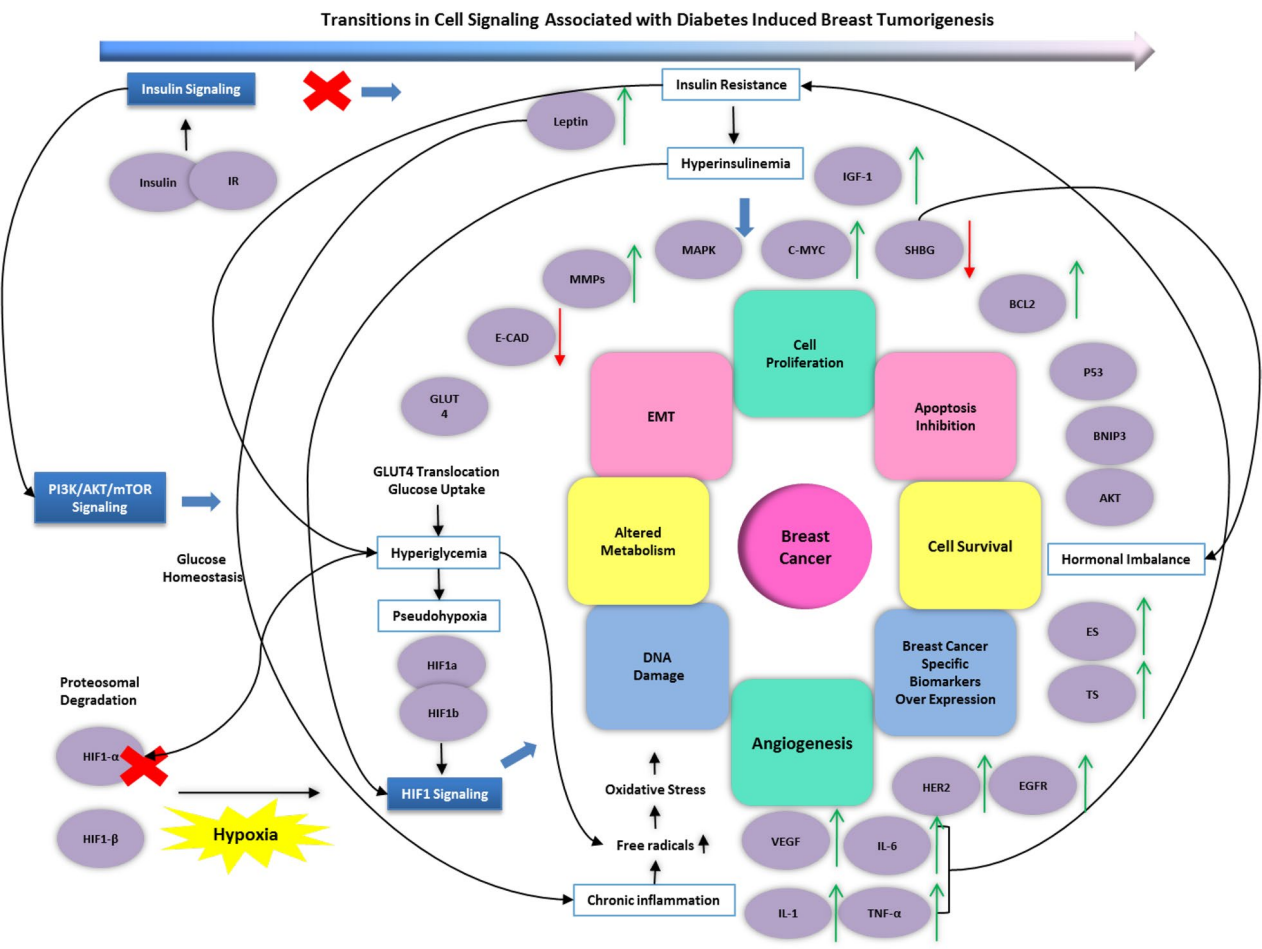
Transitions in cell signaling associated with diabetes induced breast tumorigenesis. HIF1 signaling not only cross-talks with Insulin but also other crucial pathways implicated in diabetes mellitus and breast cancer. Key players (identified by purple circles) along with the states contributing to their regulation (blue border-white box) and in turn feed into major signaling pathways (blue box) involved in the diabetes to breast cancer transition [44, 125–128]

Hence, key players of the diabetes- breast cancer crosstalk include insulin and its binding to insulin receptor which triggers the insulin pathway, and which subsequently leads to the activation of PI3K-AKT and MAPK pathways, and HIF1 signaling via mTOR pathway [124]. Hypoxia is also shown to activate the AKT pathway [106]. HIF1 signaling up regulates GLUT1 and GLUT3 which in turn regulates metabolism, VEGF, which controls angiogenesis and hence contributes to cell survival, and also lead to the activation of c-Myc and IGF2, along with other genes which promote cell proliferation as shown in Fig. 4.

An intricate crosstalk between HIF1 signaling and other key players including breast cancer specific markers estrogen and HER2, and other cell survival and proliferation markers may mediate the hallmarks of transition from diabetic to comorbid state.

## Hallmarks of diabetes and breast cancer

### Hyperglycemia and its complications

Hyperglycemia is a term used for high blood glucose level, caused either by a lack of insulin secretion or its inaction [129]. It is a characteristic of diabetes, but has also been reported in non-diabetic acute illness. It is also considered a physiological response to inflammation, and leads to an increased production of cytokines such as NF-κB and c-reactive protein (CRP) [130].

Hyperglycemia is a risk factor for cancer progression as it has shown to lead to tumor growth. However, the underlying molecular mechanism is not fully understood, yet there are several ways through which hyperglycemia contributes to tumor progression. This includes the up-regulation of glucose transporters such as GLUT1 and GLUT3, and growth factors, which contribute to cancer cell proliferation. Glucose metabolism leads to advanced glycation products (AGEs) and their interaction with receptors (RAGEs), ultimately leading to increased oxidative stress, contributing to DNA damage and genomic instability. Hence the role of hyperglycemia is well established in linking the metabolic nature of both diabetes and breast cancer.

### Hypoxia induction and its aftermath

Hypoxia is the state of inadequacy of oxygen in a tissue due to any reason [131]. Increasing evidence highlights its role in multiple diseases and metabolic disorders such as diabetes [132], and cancer [133, 134]. Hypoxia, along with other conditions such as inflammation, ER-stress and mitochondrial dysfunction, are all associated with insulin resistance, which is a characteristic of type 2 diabetes.

Notably, it is one of the major contributing factors to tumor malignancy. The extent and time of exposure to hypoxia, partially determines the cancer cell’s response to it; whether it leads to cell death or cell survival via triggering autophagic pathways. Prolonged hypoxia leads to increased ROS production, which promotes tumor cell survival and progression.

### The hypoxia-hyperglycemia axis

Hyperglycemia reportedly induces hypoxic conditions and generation of mitochondrial ROS. Low oxygen levels stabilize HIF1-α subunit, which then binds with the beta subunit to mediate cell's coordinated response to hypoxia.

It is already known that hyperglycemia and hypoxia interact via common mechanisms to cause complications in diabetic patients. Both the conditions have shown to promote glycolysis, however via independent pathways and these effects are shown to be additive in causing diabetic retinopathy [135]. Hypoxia also reportedly decreases insulin signaling in adipocytes [136].

Additionally, high glucose level in breast cancer patients has been associated with elevated IGFBP2 levels and subsequently with chemo-resistance, highlighting the role of IGFBP2 in modulating a cancer cell’s response to chemotherapy. Since T2DM is characterized by hyperglycemia, the potential role of IGFBP2 in T2DM induced breast cancer, and particularly as a therapeutic target to increase chemo-sensitivity of breast cancer cells seems plausible. Under the hypoxia-hyperglycemia equation, hypoxic conditions have shown to correspond with reduced IGFBP2 levels, consequently negating the chemo-resistant effects of hyperglycemia [137]. However, further research is necessary to fully understand the molecular nature of IGFBP2’s role in chemo-resistance, particular within the hypoxia-hyperglycemia crosstalk.

Furthermore, hyperglycemia may also activate HIF1 via mTOR signaling [138]. Hence, it is crucial to understand the crosstalk fully, specifically the impact of hyperglycemia on HIF1 pathway in breast cancer cells, in order to determine the molecular basis of diabetes-breast cancer link. This may be possible by applying a mechanistic approach to identifying potential biomarkers such as HIF1 that could crucially determine and regulate mutual aberrations in both diabetes and breast cancer and how pre-morbiditic diabetic state may progress into a mammary cancerous state.

### Hyperinsulinemia

Increased phosphorylation of IRS proteins prevents tyrosine phosphorylation leading to insulin resistance [139], which leads to compensatory hyperinsulinemia. Moreover, high insulin level is independently associated with breast carcinogenesis and progression, and this is supported by reports of insulin overexpression in breast cancer cells [140]. Once it binds to its receptor, it activates cell-signaling leading to cell survival, increased glucose uptake, mitogenesis, cell proliferation, invasion, and metastasis [30]. Besides, its direct mitogenic effects, it induces the expression of other factors including IGF-1 and HIF-1 [141], and increases bioavailability of estrogen [142].

### Chronic inflammation

Increased production of cytokines and adipokines particularly by the adipose tissue in diabetic state may provide a favorable microenvironment for breast tumor growth. Specifically, interleukin-6 (IL-6), may lead to the activation of the JAK-STAT pathway, which not only enhances cell survival and proliferation, but also inhibits host anti-tumorigenic immune response [143]. Interestingly, HIF1 yet again plays a central role in mediating a connection between inflammatory response and carcinogenesis. It promotes the recruitment of immune cells and positively regulate the function of pro-tumorigenic inflammatory response cells [144], forming yet another molecular bridge between T2DM and breast cancer.

### Hormonal imbalances

Besides the implication of the insulin/IGF-1 axis, diabetes involve the dysregulation of other hormones, which implicate HIF1 signaling and may promote breast carcinogenesis such as the adiponectin-leptin duo. In patients with obesity associated diabetes, elevated leptin levels lead to increased aromatase and estrogen production, release of pro-inflammatory cytokines, cell proliferation, migration and invasion [145]. This coupled with low levels of anti-tumorigenic adiponectin facilitate tumor progression [146].

## HIF1’s regulation of the multilayered, relationship between T2DM and breast cancer

### Multilayers within the T2DM-BC association

The complex nature of the association between diabetes and breast cancer becomes evident, as T2DM not only affects breast cancer risk but also its prognosis, besides an independent link between diabetic treatment and breast cancer risk and prognosis. Conversely, breast cancer has also been associated with the development of T2DM, and there are reports of cancer medication inducing T2DM characteristics in patients, as detailed in this section.

#### T2DM and breast cancer risk

T2DM is associated with a 20% increased breast cancer risk. However, the discrepancy in the status of HIF1 expression in diabetic patients needs to dealt with further research to elaborate on its potential diagnostic and predictive role as a biomarker for breast cancer patients with diabetes.

There are reports of associating diabetes with breast cancer, positively correlating it with ER expression negative status, however the results are conflicting, and further studies are required to clarify on this [66].

#### T2DM and BC prognosis

Breast cancer patients with pre-existing diabetes have an overall worse prognosis and decreased survival. Literature has shown that the therapeutically enhanced expression of HIF1 in diabetic patients may help alleviate diabetic complications. However, there are also reports of HIF1 targeting as a potentially effective strategy for targeting HIF1 mediated insulin resistance and treating diabetes [147]. This disparity in the comprehension of the role of HIF1 in diabetic pathogenesis raises the question of whether HIF1 targeting may be a potentially effective strategy in treating breast cancer complicated with diabetes. Although the significance of HIF1 targeting in breast cancer is well established, and is associated with overall improved survival, however, depleting HIF1 in breast cancer patients with diabetes may affect diabetic complications associated mortality in these patients, and may prove as an added health concern.

#### T2DM treatment and BC

As previously discussed, diabetic treatments report an independent association with breast cancer incidence and prognosis. The therapeutic use of insulin and its analogues in diabetic patients was shown to correlate with a higher incidence of breast cancer [71, 148], however these conflict with another study reporting the inhibitory effect of insulin on tumor growth [149]. Metformin targets PI3K-AKT pathway and AMPK reduces glucose level in diabetic patients, and is shown to exhibit anti-tumorigenic properties [150, 151]. For other glucose lowering drugs such as sulfonylureas and glinides, there is yet again inconsistency in published literature, hence requiring further investigation.

#### BC treatment and T2DM

Conversely, there are certain breast cancer treatments including temsirolimus and everolimus, which target tyrosine kinases, and lead to the development of hyperglycemic condition characteristic of diabetes [152]. Additionally, anti-neoplastic glucocorticoids are also prescribed as adjuvant therapy, which induce diabetes [150].

Herbal extracts such as the common sage (Salvia Officialis) extract have also reported anti-hyperglycemic, anti-inflammatory and anti-proliferative activities and hence may potentially be effective against treating breast cancer complicated with T2DM or diabetes induced breast cancer [153]. However, future studies are required to further elucidate on its therapeutic efficacy in co-morbid state.

### HIF1 expression in type 2 diabetes mellitus

There are numerous reports of hyperglycemic conditions destabilizing HIF1-α level, associating it to diabetic complications such as impaired wound healing [154–156]. This is consistent with the report of HIF1-α expression up-regulation in diabetic mice ameliorating wound healing and angiogenesis, elucidating on the prognostic advantage of restoration of HIF1-α expression in diabetic state. Additionally, down-regulated HIF1 expression in diabetic foot ulcer, exposed to hypoxic but not hyperglycemic condition, was also reported [157]. The status of HIF1 expression, directly regulating VEGF level, is also associated with vasculature response, and its impairment may translate into vasculature-associated complications in diabetic patients [158]. The effect of hyperglycemia on HIF1-α level was further investigated and it was found that glucose affected HIF1 expression only under hypoxic conditions in human dermal fibroblasts (HDF).

Furthermore, the knockdown of HIF1 in adipocytes resulted in increased insulin secretion leading to increased glucose tolerance and amelioration of insulin resistance, establishing its potential as a promising T2DM therapeutic target [159, 160]. This is supported by the role HIF1 plays in inflammation response leading to insulin resistance in adipose tissue [147].

Contrary to this, HIF1 is stabilized by both insulin and IL-1, which are overexpressed in diabetes under normoxic condition [138, 161, 162]. PI3K-AKT pathway activated by hypoxia and crucial to insulin signaling is also implicated in the stabilization and accumulation of HIF1, which in turn has a positive impact on insulin sensitivity and glucose metabolism in skeletal tissue [163]. Hence, there may be tissue specific signaling determining the influence of HIF1 in diabetic state and further research may shed light into the cell type specific expression status of HIF1 in T2DM.

### HIF1 expression in breast cancer subtypes

Further broadly categorizing breast cancer subtypes as outlined in Fig. 2, based on hormone expression status, three main categories emerge, hormone expressing, HER2 expressing and triple negative breast cancer (TNBC), with the TNBC subtype being most aggressive. A study conducted in 2019, reported a higher TNBC and HER2 expressing breast cancer specific increased risk in diabetic patients, particularly for TNBC, which expresses higher IGF level [66], along with long-term metformin usage particularly associated with increased odds of developing TNBC.

Relating to the reported HIF1 expression across these breast cancer subtypes, may provide insight into the subtype specific potential of HIF1 as a therapeutic target. Tumor hypoxia and HIF1 expression is associated with tamoxifen resistance and overall poorer prognosis in estrogen receptor positive breast cancer [164]. HER2 overexpressing breast cancer cells reportedly stabilized HIF1 levels under normoxic conditions, highlighting at its role in HER2 breast cancer specific signaling [165]. Similarly, increased HIF1 expression in TNBC is associated with more aggressive phenotype, hence necessitating the therapeutic targeting of HIF1 to enhance disease prognosis [166].

### Breast cancer subtype specific signaling

The molecular signaling underlying different breast cancer subtypes may implicate common and differential signaling pathways. The up-regulation of insulin and IGF is particularly higher in TNBC relative to those in the estrogen responsive cells, and which may then lead to HIF1 activation [167]. Similarly, cytokines such as IL-6 and IL-8 are implicated in tumor growth and evading apoptosis in TNBC but not in ER-positive breast cancer cells [168].

Hormone expressing subtypes express ER and/or PR, HER2 enriched breast cancer overexpress HER2 whereas TNBC do not express ER, PR and HER2. Estrogen, HER2 and EGFR signaling pathways once turned on in different breast cancer subtypes, activate common downstream signaling pathways such as PI3K-AKT and MAPK/ERK pathways which then mediate the hallmarks of cancer.

### HIF1-α’s mediation of T2DM-BC Association

Increased HIF1 expression is associated with overall poorer survival in breast cancer patients [169], and several studies directly or indirectly implicate HIF1-α in the diabetes-breast cancer crosstalk. A study conducted in 2015, reported HIF1 mediated advancement of breast metastasis in comorbid patients [170], however the article cannot be fully reviewed, as it was excluded based on language discrepancy. Hence, to date, to the best of our knowledge, there has been very limited prior experimental study designed to potentiate the role of HIF1-α in diabetes-breast cancer crosstalk. Published in 2017, a study reported the induction of HIF1 expression by hyperinsulinemia mediated inhibition of HIF1-α ubiquitination, in estrogen receptor positive breast cancer cells derived from breast tumor in T2DM mice [171]. HIF1, once activated, may promote tumorigenic activities leading to the initiation and progression of breast cancer in subjects with diabetes, hence potentiating the role of HIF1-insulin axis in T2DM-BC crosstalk, since, both T2DM and breast cancer are characterized by hypoxia state.

Furthermore, insulin also leads to the activation of leptin, an obesity related hormone, already known to associate with breast cancer progression via transcription factors including HIF1 and its crosstalk with PI3K-AKT and ERK1/2 pathways [172, 173].

Additionally, tumor microenvironment consists of cells including adipocytes [174], which synthesize bioactive molecules including growth factors, estrogen, and leptin, exposing mammary tissue to pro-tumorigenic factors [175]. Leptin activates the JAK-STAT breast cancer pathway leading to c-MYC and BCL2 expression mediated cell growth and proliferation [176, 177], whereas estrogen may regulate the development of estrogen dependent breast cancers [178]. Leptin also leads to HIF1 mediated increased expression of aromatase, which is required for estrogen signaling.

Obesity may also serve as a factor for breast cancer development, as the addition in adipose tissue composing the breast may promote the development of hypoxic conditions, inflammatory response and insulin resistance [179].

**Fig. 5:**
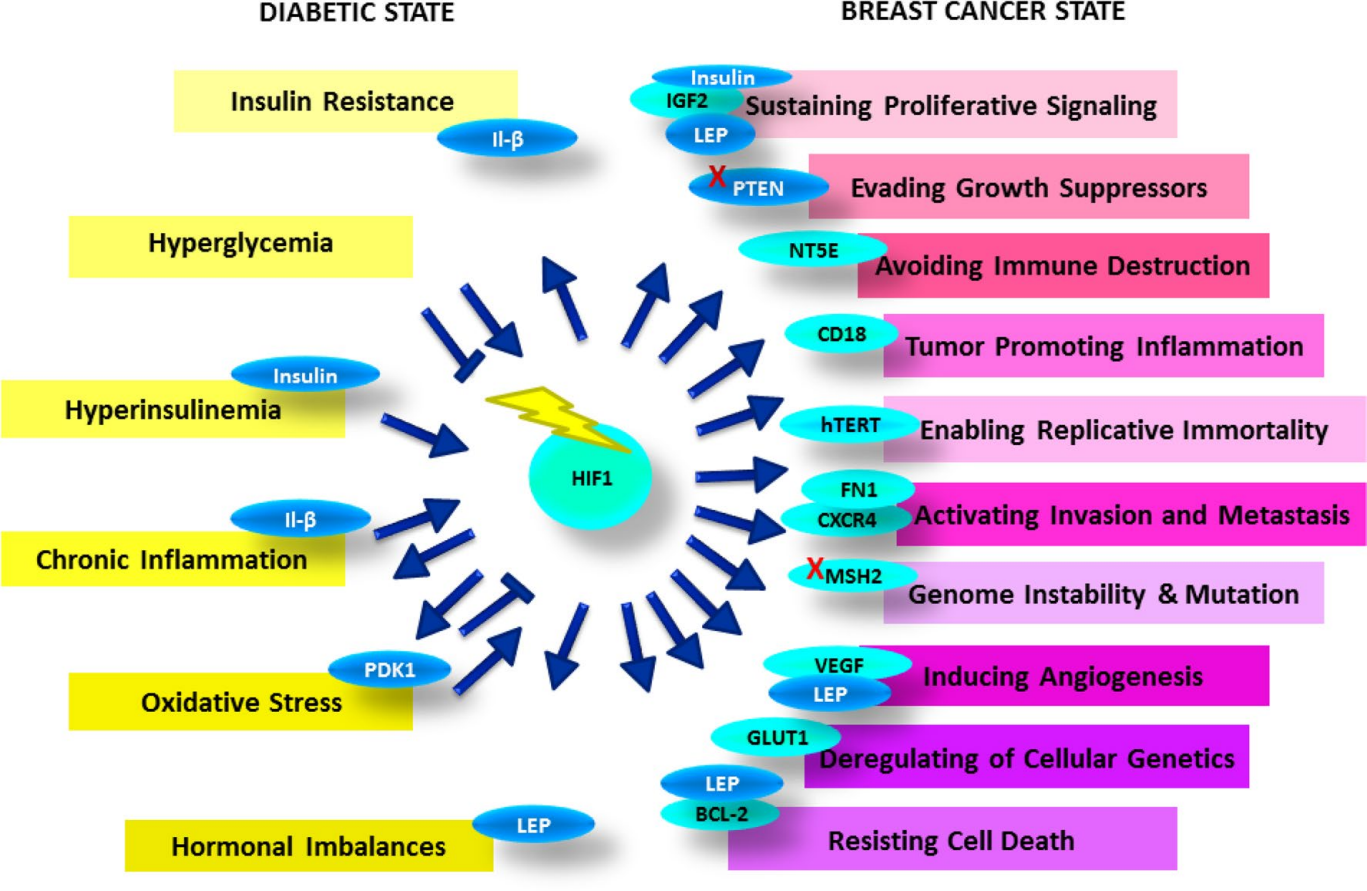
HIF-1 relaying crosstalk between T2DM and breast cancer hallmarks. It is regulated by hyperinsulinemia, hyperglycemia and other hallmarks of diabetes and once activated, it mediates insulin resistance and other features of T2DM and hallmarks of breast cancer

Hence, HIF1-α is intricately involved in mediating phenotypic characteristics of T2DM and breast cancer, and the T2DM-BC crosstalk, as annotated in Fig. 5.

The molecular alterations within the diabetic state may stabilize or destabilize HIF1 level. Once HIF1 is activated, it feedbacks into mediating diabetic hallmarks such as insulin resistance, oxidative stress and chronic inflammation. It relays the molecular crosstalk to mediate the development of hallmarks of cancer, directly via its target genes such as IGF2, CD18, VEGF, FN1, GLUT1 and BCL2 and indirectly by inducing other factors such as insulin, leptin and negatively regulating PTEN.

Additionally, several studies discuss the role of various therapeutic agents targeting diabetes and breast cancer, while also implicating HIF1-α. Recently, there has been an emerging focus on studying the role of metformin, an anti-diabetic drug, as an anti-cancer therapeutic avenue [180]. It is being pursued for its potential to be repurposed as an anti-cancer drug [179], and associated with an overall reduced cancer risk in diabetic patients. It was shown to induce p-AMPK mediated prolyl hydroxylases (PHDs) expression in cancer-associated fibroblasts, a major constituent of the tumor microenvironment, leading to HIF1 inhibition and subsequently decreased breast cancer invasion. Hence, metformin’s role in targeting pro-tumorigenic reprogramming in tumor microenvironment especially by modulating tumor-stromal crosstalk may be studied further. However, this may lead to the question of whether metformin would be an effective therapeutic strategy for co-morbid patients already expressing low HIF1 levels, in lieu that it may further aggravate diabetic complications. Hence, further research is necessary to understand this.

Additionally, a microRNA, miR-18a has been implicated in diabetes- breast cancer crosstalk and it reportedly target HIF1-α [181, 182], and YC-1 is a specific HIF1-α signaling inhibitor by blocking HIF1-α synthesis [183].

Another study, conducted in 2012, showed the increased expression of HIF1 in co-morbid patients, and which significantly decreased in response to metformin treatment [184]. Where hyperglycemia is shown to destabilize HIF1 levels, there are also reports of targeting HIF1 leading to amelioration of obesity and insulin resistance, further elaborating on its potential as therapeutic agent [160].

Metformin, derived from biguaide, is a first line T2DM oral treatment, as prescribed by America Diabetes Association (ADA) [185]. Recent evidence is emerging on its potential role as an anti-cancer therapeutic; however the underlying mechanism for this property is not fully understood. It is also shown to inhibit HER2 mediated cell proliferation and angiogenesis via targeting of HIF1 and its downstream transcriptional activation of VEGF, a master regulator of angiogenesis [186].

Furthermore, metformin is also reported to promote AMPK signaling mediated apoptosis in breast cancer cells and white adipose tissue endothelial cells [187], which have been shown to play a cooperative role in breast cancer angiogenesis and metastasis, hence modulating both the tumor and its microenvironment, specifically in TNBC and HER2 positive breast cancer cells.

Another anti-diabetic drug shown to possess anti-tumorigenic property is rhaponticin (RA), derived from medicinal herbs [188]. It was shown to suppress angiogenesis, metastasis and resistance to apoptosis in breast cancer cell line MDA-MB-231, derived from TNBC, via targeting the HIF1 signaling pathway.

Additionally, dacosahexaenoic acid is a type of omega-3 polyunsaturated fatty acid, which is reported to not only be effective for diabetes, but also for cancer with its anti-proliferative, anti-angiogenesis, anti-invasion, anti-metastatic and pro-apoptotic properties, via negatively regulating HIF1-α levels, leading to downregulation of glycolytic enzymes in breast cancer cells [189].

### Targeting HIF1 in T2DM-breast cancer patients dilemma

**Fig. 6:**
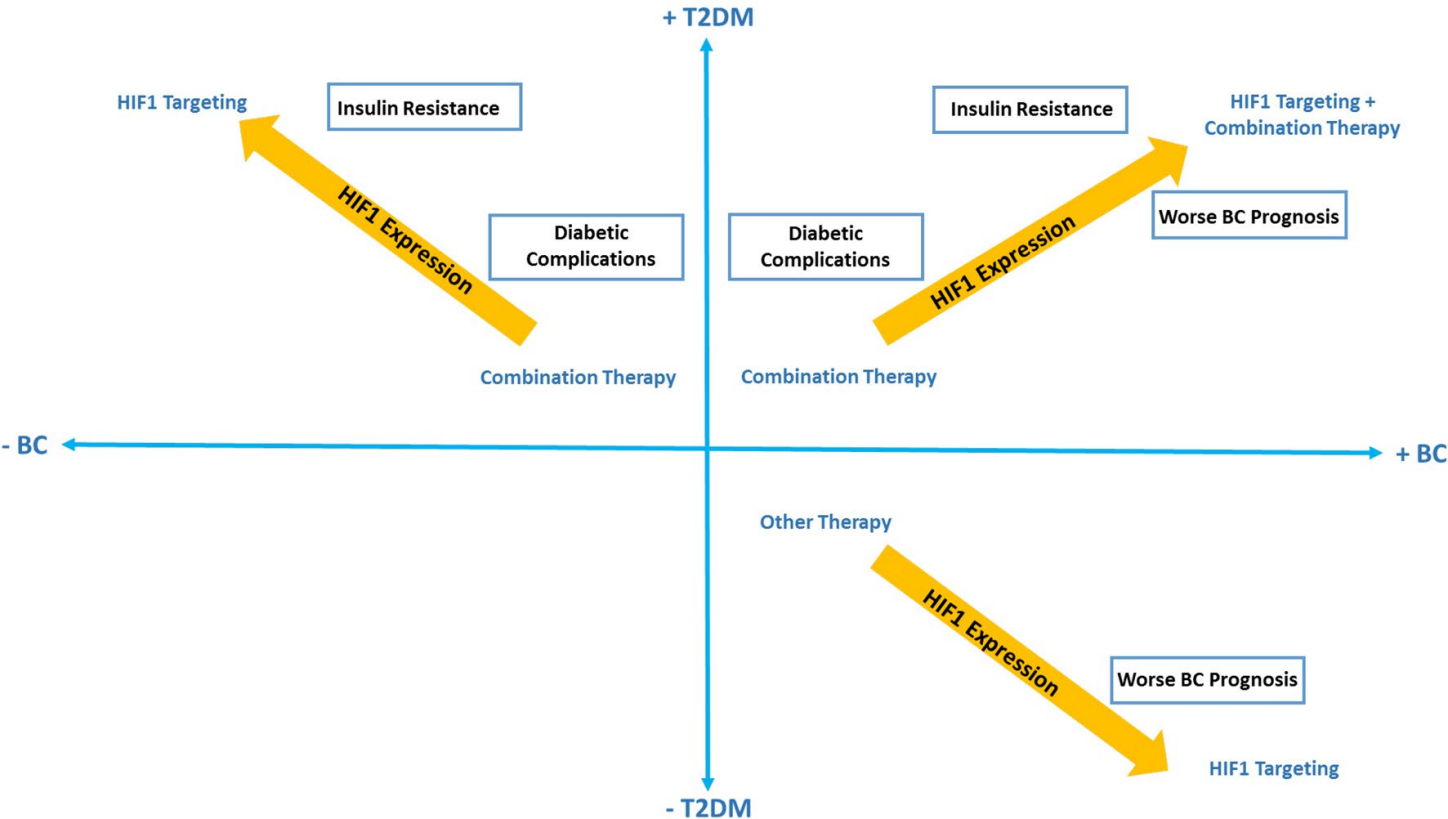
Proposed scheme for devising a therapeutic strategy for T2DM-breast cancer patients

Disparity in findings reporting HIF1 expression in diabetic patients creates a need for further research on its cell type specific expression, to enable HIF1 expression based patient selection in breast cancer patients with T2DM before opting for HIF1 targeting as a therapeutic strategy. To this end, we propose a scheme as shown in Fig. 6, suggesting considerations for devising therapeutic strategy for diabetic, breast cancer and comorbid patients. While targeting of HIF1 may tackle with breast cancer and diabetes associated worse prognosis, the application of combination therapy such as the addition of VEGF expression enhancers may prevent the implications of diabetic complications that may accompany the therapeutic down-regulation of HIF1 in patients with both T2DM and breast cancer.

HIF1 targeting in co-morbid patients may complicate diabetic prognosis further, hence combination therapy to treat diabetic complications alongside HIF1 targeting may be necessary. Similarly, for high HIF1-α expression in T2DM patients at risk of developing BC, HIF1 targeting may prove a promising therapeutic strategy, whereas for BC patients at risk of developing T2DM, HIF1 targeting may potentially alleviate disease prognostic outcome. However, in patients deficient in HIF1-α expression, other therapeutic strategies may need to be opted for.

## Initiation and progression of breast cancer

The transformation of a normal cell into a malignant cancer cell involves a series of complex changes including initiation, promotion and progression [190]. There are numerous factors that could trigger and facilitate this transformation which include but are not limited to diet, prolonged exposure to carcinogens and obesity [191]. Increasing evidence has been associating diabetes, particularly T2DM with cancer risk, development, prognosis and even treatment [192] and T2DM is being established as a risk factor for cancer development. Hyperglycemia related complications are increasingly prevalent in cancer patients indicating at the role it may play in progressing cancer.

At molecular level, hyperglycemia and insulin mediated HIF1 signaling activation favors the development of hallmarks of cancer with the transforming cell. HIF1 target genes such VEGF promotes angiogenesis, GLUT1 and PDK-1 mediates metabolic shift to anaerobic metabolism [193], BCL2 regulates anti-apoptotic behavior and MMPs orchestrate epithelial to mesenchymal transition. Within this tumor promoting micro-environment, the build-up of ROS triggers DNA damage [194], and coupled with this, HIF1 also mediates inflammation promoting activities [195, 196]. In synergy with all these molecular changes, insulin-IGF duo pushes the cell toward proliferation.

## Conclusion

Hypoxia inducible factor 1 is a master regulator of hypoxia mediated cell response including alterations in the metabolic pathways particularly involving glucose uptake and its metabolism, and can be activated under non-hypoxic conditions. Its mediation of hallmarks of diabetes and breast cancer highlights its centrality to the molecular interplay between these two complex diseases and in governing the associated molecular players establishing the molecular connections between diabetes and breast cancer. This potentiates its relevance as a biomarker with prognostic and therapeutic significance, however there is very limited research done on this and further experimentation is required to fully comprehend the role of HIF1 in comorbid state.

Furthermore, it is also paramount to consider the implication of targeting HIF1 expression, which, although it may alleviate prognosis of breast cancer, and diabetes associated insulin resistance, yet may still translate into diabetic complications particularly those vasculature in nature, and worsen the overall survival outcome. Hence, there is a need to consider expression levels of potential prognostic markers such as HIF1 in comorbid patients, to ensure a more effective therapeutic strategy against breast cancer complicated with diabetes. Combination therapy such as VEGF expression enhancers may be prescribed to patients selected for HIF1 targeting therapy, to prevent diabetic complications associated mortality in breast cancer patients. While, targeting HIF1 and other associated potential biomarkers may in fact reduce mortality associated with breast cancer in diabetic patients, eventually alleviating the socioeconomic burden of diabetes- breast cancer association on global health.

## Data Availability

Not applicable.

## Abbreviations

HIF 1: Hypoxia inducible factor 1
T2DM: Type 2 diabetes mellitus
TNF-α: Tumor necrosis factor- alpha
IGF: Insulin-like growth factor
PI3K: Phosphatidylinositol-3-kinase
mTOR: The mammalian target of rapamycin
T1DM: Type 1 diabetes mellitus
IRS: Insulin receptor substrate
MAPK: Mitogen-activated protein kinase
PHD: Prolyl hydroxylase
FIH1: Factor inhibiting HIF-1
ERK: Extracellular-signal-regulated kinase
IL: Interleukin
GLUT: Glucose transporter
NF-κB: Nuclear factor kappa-light-chain-enhancer of activated B cells
GSK: Glycogen synthase kinase
MMP: Matrix metallo-peptidase
BNIP3: BCL2/adenovirus E1B 19 kDa protein-interacting protein 3
VEGF: Vascular endothelial growth factor
AMPK 5': AMP-activated protein kinase
STAT3: Signal transducer and activator of transcription 3
GF: Growth factor
MYC: Master regulator of cell cycle entry and proliferative metabolism
BRCA1: Breast cancer protein 1
COX2: Cyclooxygenase 2
EPO: Erythropoietin
HK: Hexokinase
LDH-A: Lactate dehydrogenase A
PKM: Pyruvate kinase muscle isozyme
MDR: Multi-drug resistance protein
OCT4: Octamer-binding transcription factor 4
E-CAD: E-cadherin
SBHG: Sex hormone binding globin
BCL2: B-cell lymphoma 2
ES: Estrogen
TS: Testosterone
IR: Insulin receptor
CRP: C-reactive protein
ROS: Reactive oxygen species
